# Seed layer technique for high quality epitaxial manganite films

**DOI:** 10.1063/1.4961228

**Published:** 2016-08-12

**Authors:** P. Graziosi, A. Gambardella, M. Calbucci, K. O’Shea, D. A. MacLaren, A. Riminucci, I. Bergenti, S. Fugattini, M. Prezioso, N. Homonnay, G. Schmidt, D. Pullini, D. Busquets-Mataix, V. Dediu

**Affiliations:** 1 CNR - ISMN, Consiglio Nazionale delle Ricerche - Istituto per lo Studio dei Materiali Nanostrutturati, v. Gobetti 101, 40129 Bologna, Italy; 2 Instituto de Tecnología de Materiales, Universitat Politécnica de Valencia, Camino de Vera s/n, 46022, Valencia, Spain; 3 Scottish Universities Physics Alliance, School of Physics and Astronomy, University of Glasgow, Glasgow, United Kingdom; 4 University of California, Santa Barbara, Electrical & Computer Engineering Harold Frank Hall, Santa Barbara, CA 93106-9560, USA; 5 Institut für Physik, Universität Halle, 06120 Halle, Germany; 6 Interdisziplinäres Zentrum für Materialwissenschaften, Martin-Luther University Halle-Wittenberg, Nanotechnikum Weinberg, 06120 Halle, Germany; 7 Centro Ricerche Fiat, 10043, Orbassano, Torino, Italy

## Abstract

We introduce an innovative approach to the simultaneous control of growth mode and magnetotransport properties of manganite thin films, based on an easy-to-implement film/substrate interface engineering. The deposition of a manganite seed layer and the optimization of the substrate temperature allows a persistent bi-dimensional epitaxy and robust ferromagnetic properties at the same time. Structural measurements confirm that in such interface-engineered films, the optimal properties are related to improved epitaxy. A new growth scenario is envisaged, compatible with a shift from heteroepitaxy towards pseudo-homoepitaxy. Relevant growth parameters such as formation energy, roughening temperature, strain profile and chemical states are derived.

Ferromagnetic manganites are a prototypical example of half metals: materials with 100% spin polarization at zero temperature. Although their application in commercial devices is limited by a relatively low Curie temperature (*T_C_* ≤ 370 K), manganites represent an excellent research model for testing spin injection into various materials and to search for pioneering device paradigms.^1–5^ For instance, they have contributed significantly to the field of organic spintronics, where almost half of the reported devices have La_0.7_Sr_0.3_MnO_3_ (LSMO) as a spin injector.^6,7^ In these and other devices, it is imperative to optimize the spin injection efficiency, which is intimately linked to the quality of the ferromagnetic layer and its interfaces.^6,8^ In many cases, however, keeping the manganite film thickness relatively low (around 12 nm) offered the best trade-off between maintaining a smooth morphology and optimizing the magnetic and transport properties.^9^

In this article, we propose a new way to increase this limit to much higher thicknesses, up to 75 nm at least, in order to push the LSMO based devices temperature operations closer to manganite T_C_. We report on epitaxial LSMO thin films deposited on SrTiO_3_ (100) (STO) by pulsed electron beam deposition in the channel spark ablation (CSA) configuration.^8,10,11^ Using atomic force microscopy (AFM) and scanning tunneling microscopy (STM), we confirm that the roughness of the films grown directly on STO depends on the deposition temperature. In particular, below a threshold deposition temperature of *T_R_* ∼ 1050 K, the growth is bi-dimensional (for thicknesses up to at least 100 nm) and the films are smoother than those grown above *T_R_*, which show a thickness induced roughening with a three dimensional growth above a certain thickness, ∼ 10 nm for STO (100) substrates. We also find that a strong magnetism is achievable only above *T_R_* where the films surfaces are rougher above 10 nm in thickness. The magnetic properties are nevertheless fully recovered, even for deposition below *T_R_*, when the film is deposited in a LSMO/*seed-layer*/STO design, where the seed layer is a film of 0.5 to 1.5 LSMO unit cells deposited at room temperature (RT) and then rapidly heated (∼ 100 °C/min) in oxygen to the film deposition temperature. Such film/substrate interface engineering preserves the film flatness characteristic of the depositions below *T_R_* and the magnetism typical of the depositions above *T_R_*.

The seed layer approach acts on a roster of parameters rather than focusing on only ne and enables a comprehensive improvement of the LSMO film quality, rather than develop just one. This approach allows the deposition of thick (75 nm at least) LSMO films with atomically flat surfaces and robust bulk and surface magnetism, even though the growth is carried out at temperatures below *T_R_*.

LSMO thin films were deposited at a rate of 0.1 ± 0.02 Å/pulse (∼0.025 unit cells per pulse, at a frequency of 6 Hz) by pulsed electron beam deposition in the CSA configuration using a commercial target; details of the deposition condition have been previously reported.^10^ The substrates were purchased from Crystal GmbH and Crystec GmbH and treated as already described.^8^ AFM measurements were conducted by a Nanoscope Multimode III in tapping mode, STM measurements were performed in an ultra high vacuum Omicron STM system with Pt/Ir tips. Magnetotransport and magneto-optic Kerr effect measurements were performed in an homemade system. X-ray diffraction (XRD) measurements were executed in a Siemens / Bruker D5000 XRD System. Scanning transmission electron microscopy (STEM) was performed with a JEOL 200cF ARM scanning instrument, equipped with a cold field emission gun and operated at 200 kV. Standard cross-sectional specimens for STEM investigation were fabricated using a FEI Dual Beam FIB Nova 200.

The AFM images in Figure 1 show the surface evolution of LSMO thin films grown on identical STO substrates (same lot) deposited at T_dep_ ∼ 1100 K without the seed layer (left column) and deposited at T_dep_ ∼ 1000 K with the seed layer (right column). Details on the substrate cleaning procedure and on film deposition have been previously reported elsewhere.^10^ Films deposited at T_dep_ ∼ 1000 K without the seed layer and films deposited at T_dep_ ∼ 1100 K with the seed layer are not shown because the morphology is not distinguishable from the ones reported here for the same T_dep_. The STO surface features terraces and 0.4 nm high steps, consistent with a single chemical termination. The LSMO surface evolution was studied by AFM on 15 nm and 35 nm thick films and by STM on 75 nm thick films. The root mean square (rms) roughness is reported in each image. The vertical scale for the films deposited at T_dep_ > *T_R_* without the seed layer increases up to 9 nm for the 75 nm thick film while for the films deposited at T_dep_ < *T_R_* with the seed layer the peak to valley roughness is confined below 1 nm – in the 75 nm film the brighter mounds are 2 nm height. It is evident that the rms roughness increases with the thickness of the films deposited directly on the STO, while it is constant, at about half unit cell, for films grown on the seed layer. The films deposited on the seed layer were grown below *T_R_* to ensure bi-dimensional growth, which is the reason for their low roughness – in the 75 nm film deposited with the seed layer approach the slightly higher rms roughness value (0.20 nm instead of 0.15 nm) is just ascribed to the higher sensitivity of the STM. In addition, the seed layer has a impressive impact on the magnetotransport properties as it will be shown later.

**FIG. 1.**
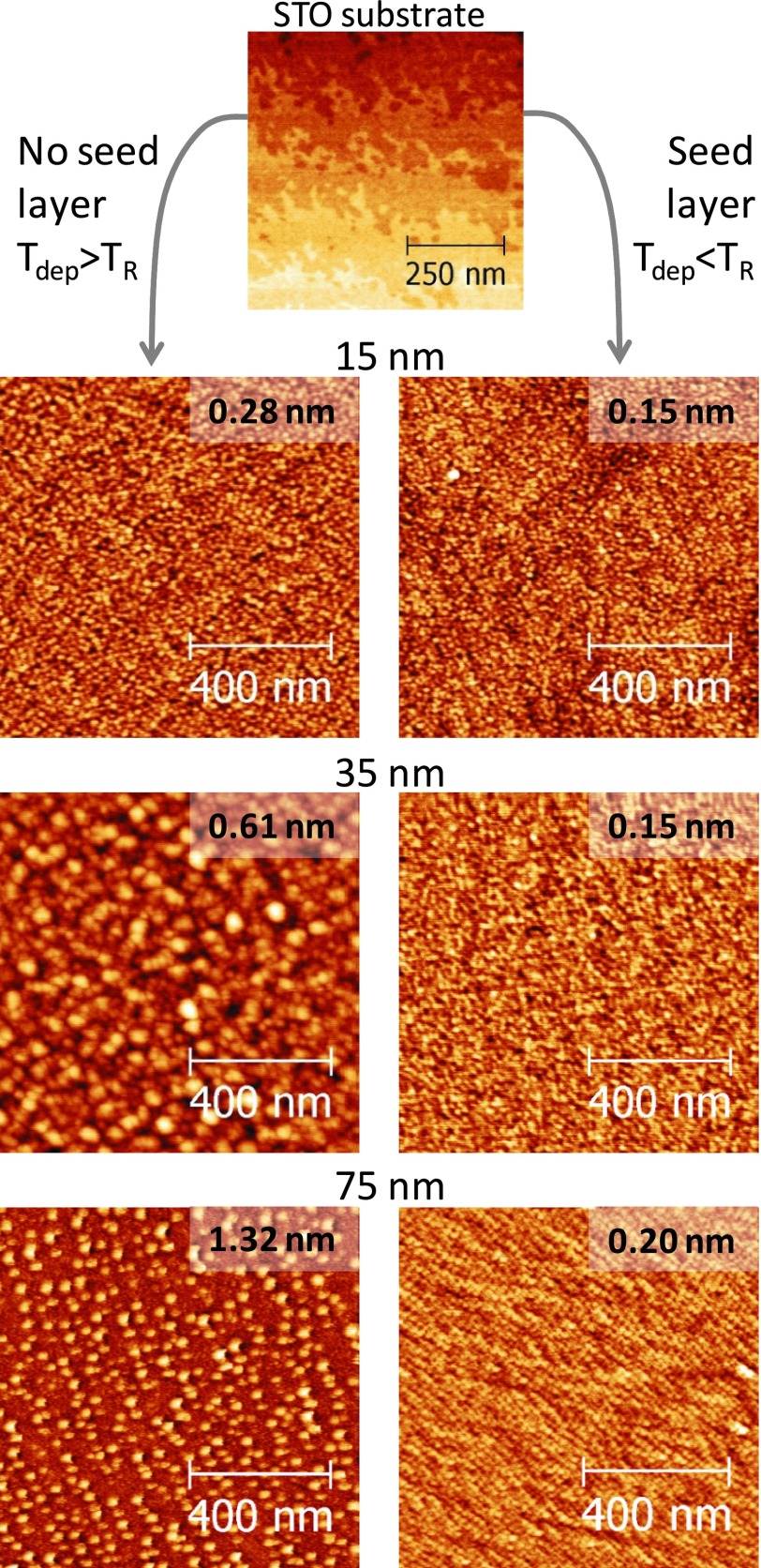
Surface evolution of the LSMO thin films, starting from the substrate (on top), for standard (left column) and engineered (right column) films. The evolution is studied by imaging films of different thicknesses (15, 35, 75 nm) as reported on the top of each row. All images were acquired using AFM, apart from the 75 nm film which was investigated by STM. The number in each image is the rms roughness.

The existence of a temperature-dependent roughening transition is well known in inorganic semiconductor epitaxy and is related to the thermodynamics of the stepped surface. Below *T_R_*, the surface evolves by the evolution of each single step, while above it, the surface is free to reorganize under the influence of infinitesimally small thermodynamic driving forces.^12,13^ In the case of crystal growth from melt or from vapor, the roughening transition takes place when:

$$\alpha =\xi \varepsilon /k_{B}T_{R}∼2.5,$$ (1)

where ξ is the ratio between the number of first neighboring atoms in the surface and in the bulk (2/3 in a cubic lattice), ε is the energy required to extract a unit cell from the crystal and put it in the phase into which the crystal is growing (melt, vapor …) and can be regarded as the enthalpy of formation, *T_R_* is the roughening transition temperature. The surface roughens when *α* ≤ 2.5.^14,15^ This picture has also been applied to metal thin films deposited by sputtering.^16^ A cube-on-cube epitaxy picture is applicable for LSMO/STO because the substrates are cubic and LSMO grows epitaxially on top. Assuming *T_R_* ∼ 1050 ± 30 K, we obtain ε ∼ 0.34 eV ± 0.01 eV per unit cell, which corresponds to 33 KJ/mol. Such a value is in fair agreement with the value of about 98 KJ/mol found for bulk LSMO obtained by solid state reaction from simple oxides.^17^ This difference can be due to three reasons. **(i)** The solid state reaction happens at thermodynamic equilibrium but additional kinetic effects are expected for the highly energetic ionized species produced in the CSA technique.^18^ **(ii)** The key steps in the formation of the epitaxial manganite films occur at the sub-unit-cell level, before the formation of a complete perovskite unit cell layer.^19^ (**iii**) The co-deposition of multiple elements leads to a variety of potential side-reactions and bonding configurations not encapsulated by the simple model of Equation (1). Most notably, the value of 33 KJ/mol is very close to the oxidation enthalpy of Mn^3+^ in Mn^4+^ (23.8 KJ/mol) in LSMO,^17^ suggesting that the oxidation processes play a crucial role in the stabilization of the LSMO phase during thin film growth.

We found a fascinating dependence of the magnetic properties of LSMO on the use of the seed layer and on the deposition temperature. Due to our setup temperature limitations, the study has been carried out on 15 nm nominal thick LSMO films having a T_C_ < 360 K. All the films presented here are metallic over the whole temperature range apart from those deposited at lower temperature without the seed layer, which show a metal-insulator transition at 335 K.

Figure 2 reports the data for these 15 nm thick LSMO. Figure 2(a) show the low field magnetoresistance, LFMR = (R_0_ – R_H_)/R_0_, versus temperature, where R_0_ is the resistance without an external magnetic field and R_H_ is the resistance with an in-plane magnetic field of 80 mT parallel to the current direction. The R(H) curves are linear in this range of fields at all the temperatures below *T_C_*. The films were deposited at T_dep_ = 1100 K (T_dep_ > *T_R_*) or at 1000 K (T_dep_ < *T_R_*) as specified by the legend. For both temperatures, we report on films deposited with and without the seed layer – for films deposited with the seed layer, it is specified in the legend. It is possible to estimate *T_C_* from a linear extrapolation to zero of the LFMR(T) plot to the right of the peak.^10^ Using this method, the highest *T_C_* value is achieved at T_dep_ > *T_R_* without the seed layer (330 K); *T_C_* drops of about 25 K when the substrate temperature is reduced below *T_R_* (ΔT in the figure) indicating that the bi-dimensional growth impairs the magnetism. Nevertheless, *T_C_* = 325 K is found where the seed layer engineering is adopted even for T_dep_ < *T_R_*. Such a variation is inside the (thickness) reproducibility of our technique.^10^ As for the role of the seed layer in films deposited at T_dep_ > *T_R_*, we notice that the effect is absent. An even detrimental effect has been observed for LSMO films deposited on NdGaO_3_ (110) (NGO) and on (LaAlO_3_)_0.3_(Sr_2_TaAlO_6_)_0.7_ (100) (LSAT) (data not shown here), suggesting that the role of the seed layer depends on the strain. Indeed the mismatch between LSMO and NGO is 0.26 % and 0.39 % (compressive strain) along the two directions of the strained LSMO unit cell, while it is -0.89 % (tensile strain) in the case of STO and less than 0.05 % in the case of LSAT substrates.

**FIG. 2.**
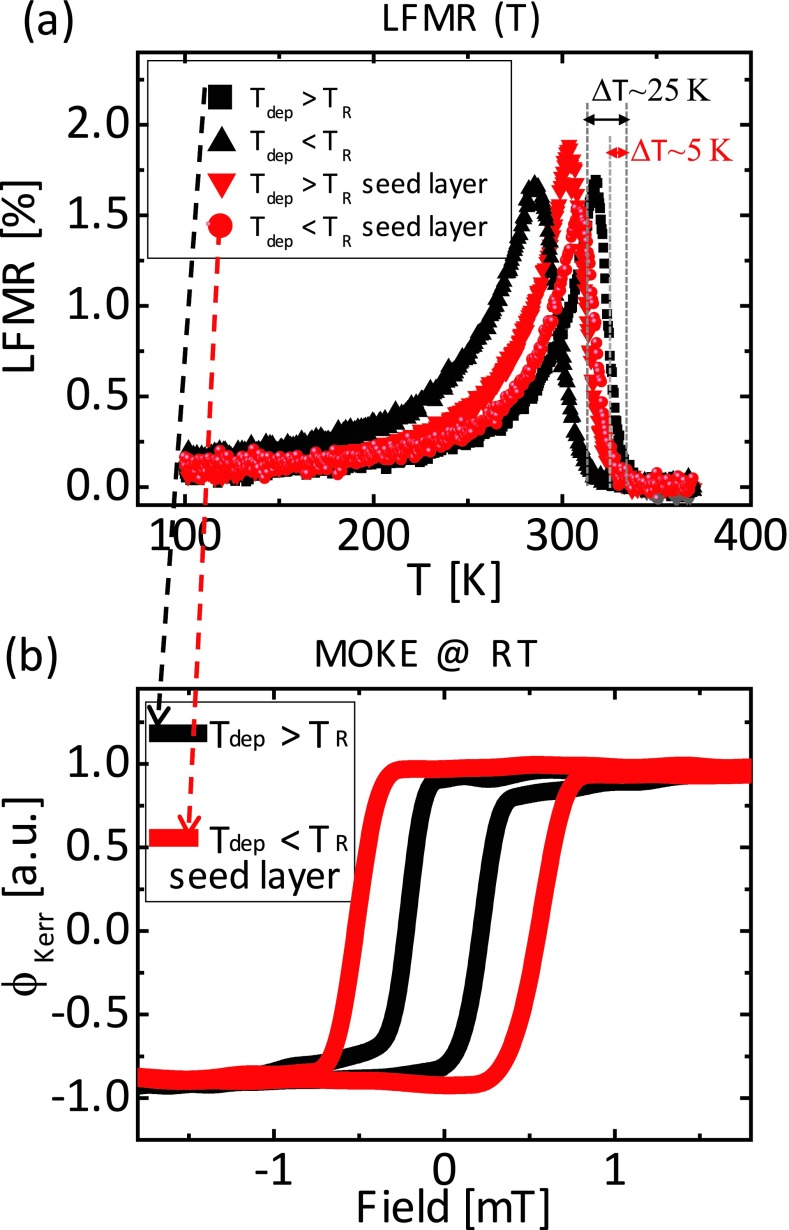
LFMR versus T (a) for 15 nm LSMO films deposited on STO above and below T_R_ and with and without the seed layer, according to the graphs legends; MOKE signal at room temperature (b) for the couples of samples with the higher T_C_.

We can conclude that the seed layer has a great influence on the magnetic properties of LSMO when the films are deposited below *T_R_*, while no (or detrimental) effect is observed when the LSMO films are grown above *T_R_*. This is consistent with the fact that the low roughness in films deposited below *T_R_* is assumed to be related to a decreased surface diffusion constant,^20^ which can be influenced by the seed layer. Differently, above *T_R_* the diffusion constant is greater, the adatoms can reach their optimal position (from a crystalline point of view) and the effect of a seed layer is minimized.

Therefore in the reminder of the paper we will compare the films deposited at T_dep_ > *T_R_* without the seed layer with the films deposited at T_dep_ < *T_R_* and with the seed layer (referred as “seed layer approach”).

Magneto-optic Kerr effect (MOKE) measurements of the same films, with the field along the easy axis of the samples, are summarized in figures 2(b). Interestingly, the films grown on the seed layer show a harder surface magnetism, as evidenced by the larger coercivity of 0.5 mT compared with 0.2 mT. The coercive field (H_C_) values appear to be not linked to the T_C_ values, defined by extrapolation to zero of the LFMR.^10^ Rather than H_C_, it is the shape of the magnetization cycle to be truly informative. Indeed the samples with the seed layer have more square cycles with the closing field corresponding to the H_C_, as single domain sample. On the contrary, the samples without the seed layer approach have closing fields higher than H_C_, which is an indication of an inhomogeneous magnetization process or multi domain sample. Thus the seed layer approach enables higher homogeneity and improved crystalline order. The geometrical phase analysis of the transmission electron microscopy data (Figure 4(e),4(f) and related comment below) support this picture.

It is interesting to correlate the magnetic properties to the structure of standard films with those grown on the seed layer, as they exhibit similar magnetic properties but different morphologies. XRD and TEM were used to collect structural information on the films. In the case of XRD, a large set of films of different thicknesses, ranging from 5 nm to 34 nm (as measured by x-ray reflectivity) was studied. The main result is shown in Figure 3, where the XRD results for (a) a 15 nm thick film deposited at *T* > *T_R_* without the seed layer and (b) a 25 nm thick film deposited at *T* < *T_R_* with the seed layer are compared. From the (003) reflection it is possible to observe that both the films started to relax, the main difference comes from the full width at half maximum (FWHM) which is higher for the film deposited without the seed layer (0.653° versus 0.212°), although it is thinner. This implies that the seed layer approach improves the degree of order in the out of plane parameter.

**FIG. 3.**
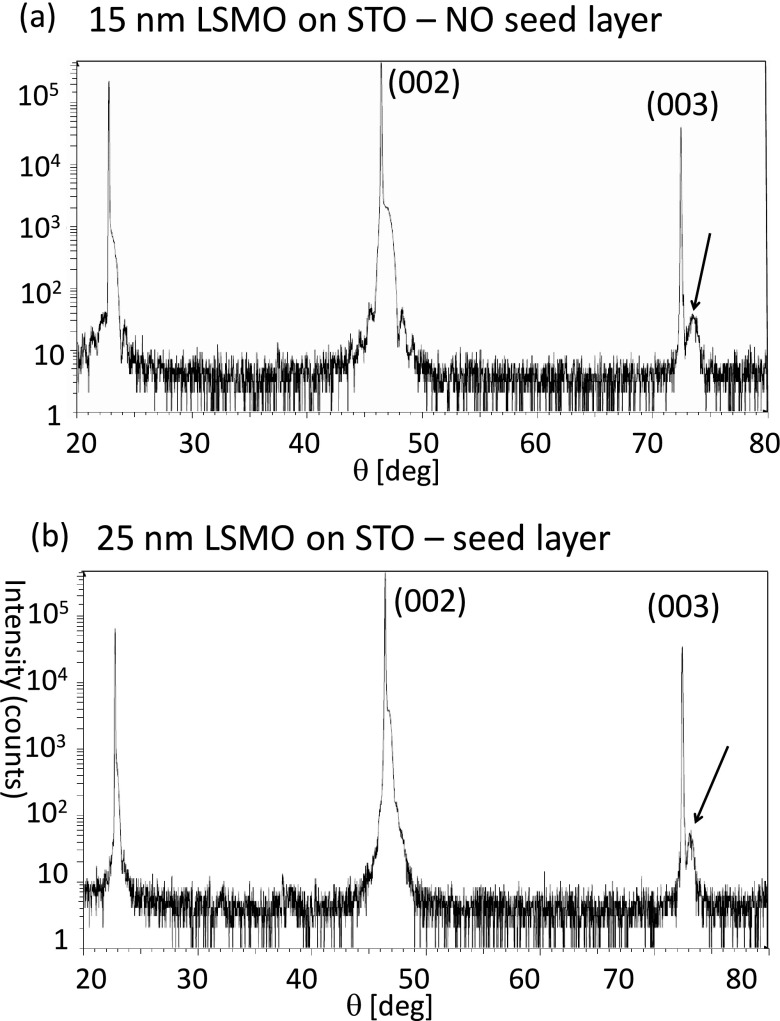
XRD characterization (a) of a 15 nm LSMO/STO film deposited above T_R_ without the seed layer and (b) of a 25 nm LSMO/STO deposited with the seed layer approach. The arrow marks the (003) reflection from the film, which is used to calculate the FWHM: 0.653 for (a) and 0.212 for (b).

These results are confirmed by scanning transmission electron microscopy (STEM) analysis, reported in figure 4. STEM images are given in figures 4(a)-4(d), where the growth direction is from right to left across the image; the protective Pt layer can be seen to the left of the LSMO. As these are high-angle annular dark field images, the contrast derives primarily from atomic number variations, and the LSMO film appears brighter than the STO substrate. In the film deposited without the seed layer, figures 4(a), 4(c), the interface with the STO substrate is somewhat diffuse, indicating a degree of intermixing at the interface as the result of the high energy deposition. This is in direct contrast to that of the sample deposited with the seed layer approach (figures 4(b), 4(d)) where a sharper interface is observed. As the seed layer was deposited at room temperature in the engineered interface sample, the thermal energy for surface diffusion is lower and the atomic species in the plume can be considered as already thermalized since the oxygen pressure in the chamber is about 4 Pa,^21^ resulting in less atomic intermixing and a sharp interface.

**FIG. 4.**
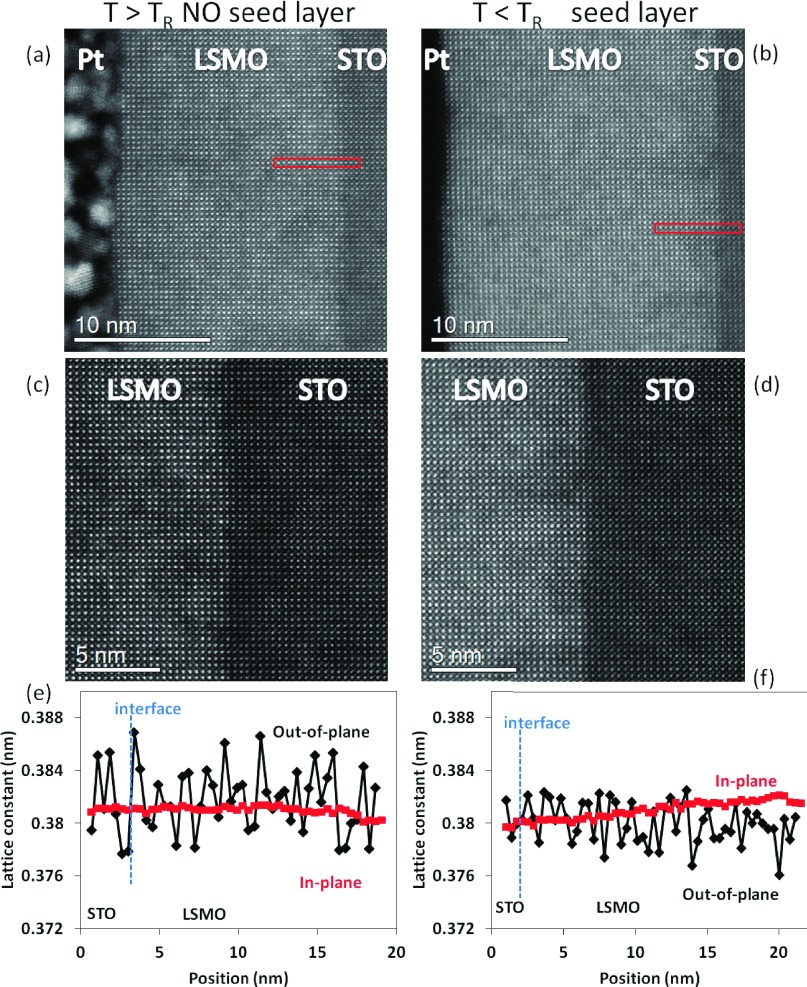
(a)-(d): STEM characterization and strain analysis of (left column) a LSMO film deposited without the seed layer approach and (right column) a LSMO film deposited with the seed layer approach. (e) and (f): GPA analysis is performed over the regions highlighted by the red rectangles in (a) and (b).

The seed layer is not noticeable at the interface in the TEM images, thus it should be part of the epitaxial system. This seems to be in contradiction with the fact that it is deposited at room temperature and hence most likely to be amorphous.^22,23^ The explanation we propose is that the amorphous seeded layer was transformed into epitaxial manganite film. Although the high growth temperature cannot be sufficient to restructure the seed layer,^24,21^ it looks realistic that the kinetic energy of the species in the plume can induce the crystallization of the seed layer during the subsequent film deposition. This also means that the material in the seed layer is utilized during the growth by the subsequent film; this available material not only changes the interaction between the early adatoms and the substrate but also acts as a material reservoir for the following film. This can explain why the out-of-plane parameters are more uniform in the films deposited with the seed layer approach (confirmed by the lower FWHM in XRD), suggesting also a different mechanism of the conservation of the unit cell volume. We can infer that the seed layer, which is a sort of material stock, moves the growth far from the intermediate proto-perovskite phase occurring when the seed layer is not used,^19^ because it provides a sufficient number of atoms to form a complete perovskite unit cell since the early atoms arrive during the subsequent deposition at T_dep_.

The geometric phase analysis (GPA) reported in Figure 4(e), 4(f), confirms that the interface engineering via the seed layer approach gives rise to excellent epitaxial growth with the substrate, comparable to or better than the optimized films deposited at T_dep_ > *T_R_*.^10^ When the seed layer approach is employed to engineer the substrate/film interface, the out-of-plane lattice parameter (i.e. along the growth direction) is more uniform across the interface and along the film growth direction, with less scattered data. The more uniform lattice constant at the substrate side in the sample with the seed layer is likely due to the seed layer itself, which imply a different film/substrate interaction at the interface, as we discuss in the next paragraph.

A real understanding of the role played by the initial material reservoir is lacking and has not yet been considered in other growth models. We suggest that the improved performance, compared with the standard films, is related to **(i)** an improved epitaxial growth, due to the departure from the proto-perovskite intermediate scenario and to a modified density of the nucleation centers, and to **(ii)** a different accommodation of crystalline defects, accompanied by local strain relaxation, in the substrate during the substrate heating after the room temperature deposition of the seed layer.^25^ This looks likely when we keep in mind that even the best single crystal STO substrates have a dislocations surface density of 10^11^ cm^−2^, which corresponds to 100 dislocations per μm^2^.^26^ A different mechanism for their relaxation is expected to have a great impact on the film properties.

**FIG. 5.**
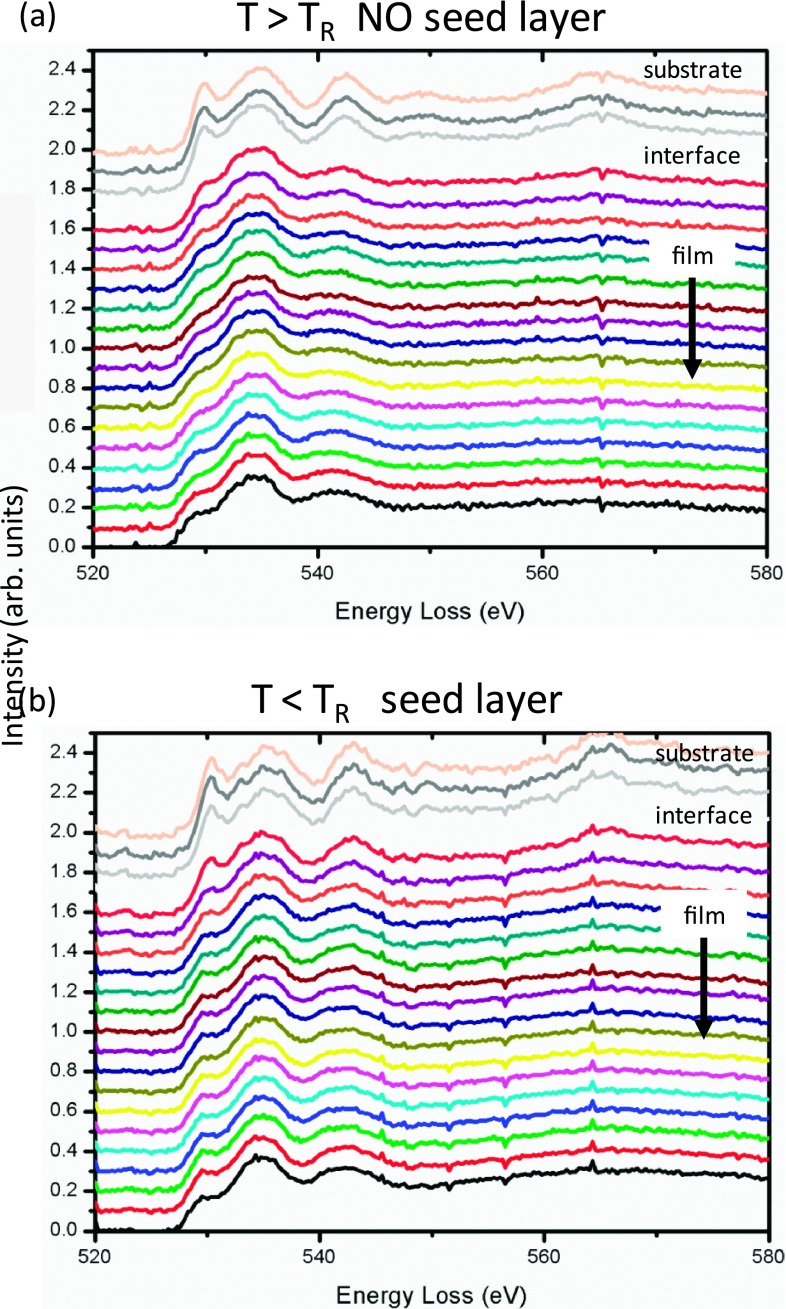
EELS analysis on the O-K edge of the sample regions highlighted in Figure 4(a), 4(b). The arrow indicates the growth direction. The main difference is in the pre-peak at 530 eV as discussed in the text.

Electron energy loss spectroscopy (EELS) on the O-K and Mn-L_2,3_ edges were performed in order to obtain chemical information about the engineered interface. While in the case of the Mn-L_2,3_ edge no obvious changes were observed, a difference in the so-called pre-peak at 530 eV^27^ was observed on the O-K edge (Figure 5). In the sample with the seed layer approach (Fig. 5(b)) the distance between the pre-peak and the main peak is about 1 eV larger. Moreover this larger split feature appears throughout the whole film. In order to understand this point it has to be stressed that the O-K edge pre-peak has a strong contribution from Mn 3d band and so it is extremely sensitive to bonding features. For instance it has been observed in the Ca analogue of LSMO (La_x_Ca_1−x_MnO_3_) that the split between the two peaks increases with the divalent atomic content (Ca or Sr).^27^ Because the samples with the seed layer approach have a greater spacing between the pre-peak and the main peak, indicating a more Sr content,^27^ the seed layer approach also enables us to fix (at least partially) the known problem that that films without seed layer are slightly deficient in Sr.^11^

In summary, we show that LSMO deposited by pulsed electron deposition at T > *T_R_* undergoes a thickness induced roughening which is suppressed by decreasing the substrate temperature, resulting in a persistent bi-dimensional growth but with weakened magnetic and transport properties. The latter are recovered, while preserving the bi-dimensional growth, by using a seed layer (0.5 to 1.5 unit cells) of LSMO deposited at room temperature, resulting in the LSMO/LSMO_(*seed*)_/STO structure. Such interface engineered films display high quality epitaxial growth and exhibit excellent magnetic properties and low roughness. Although a possible growth mechanism responsible for this improvement is proposed, many important questions remain open, especially considering the quantitative description. We believe that engineering the interface by seed layers represents a powerful tool for the simultaneous control of the film properties and roughness in various complex oxides.
